# A deep learning approach for Named Entity Recognition in Urdu language

**DOI:** 10.1371/journal.pone.0300725

**Published:** 2024-03-28

**Authors:** Rimsha Anam, Muhammad Waqas Anwar, Muhammad Hasan Jamal, Usama Ijaz Bajwa, Isabel de la Torre Diez, Eduardo Silva Alvarado, Emmanuel Soriano Flores, Imran Ashraf

**Affiliations:** 1 Department of Computer Science, COMSATS University Islamabad, Lahore, Pakistan; 2 Department of Computer Science, Government College University, Lahore, Pakistan; 3 Department of Signal Theory, Communications and Telematics Engineering, Unviersity of Valladolid, Valladolid - Spain; 4 Universidad Europea del Atlántico, Santander, Spain; 5 Universidad Internacional Iberoamericana Arecibo, Puerto Rico, Puerto Rico, United States of America; 6 Universidade Internacional do Cuanza, Cuito, Bié, Angola; 7 Universidad Internacional Iberoamericana Campeche, México; 8 Fundación Universitaria Internacional de Colombia Bogotá, Bogotá, Colombia; 9 Department of Information and Communication Engineering, Yeungnam University, Gyeongsan, Korea; University of Sargodha, PAKISTAN

## Abstract

Named Entity Recognition (NER) is a natural language processing task that has been widely explored for different languages in the recent decade but is still an under-researched area for the Urdu language due to its rich morphology and language complexities. Existing state-of-the-art studies on Urdu NER use various deep-learning approaches through automatic feature selection using word embeddings. This paper presents a deep learning approach for Urdu NER that harnesses FastText and Floret word embeddings to capture the contextual information of words by considering the surrounding context of words for improved feature extraction. The pre-trained FastText and Floret word embeddings are publicly available for Urdu language which are utilized to generate feature vectors of four benchmark Urdu language datasets. These features are then used as input to train various combinations of Long Short-Term Memory (LSTM), Bidirectional LSTM (BiLSTM), Gated Recurrent Unit (GRU), CRF, and deep learning models. The results show that our proposed approach significantly outperforms existing state-of-the-art studies on Urdu NER, achieving an F-score of up to 0.98 when using BiLSTM+GRU with Floret embeddings. Error analysis shows a low classification error rate ranging from 1.24% to 3.63% across various datasets showing the robustness of the proposed approach. The performance comparison shows that the proposed approach significantly outperforms similar existing studies.

## Introduction

Recent advancements in technologies and social media have led to the explosion of data that is being exploited to perform natural language processing (NLP) tasks to explore new research dimensions and gain useful insights. Named Entity Recognition (NER) is one of the leading research areas in the NLP domain that extracts the named entities (NEs) from that data and classifies them into a predefined set of categories [1]. NER plays a crucial role in many NLP applications including question answering [2–4], query auto-completion systems [5–7], entity linking [8–10], and search engines [11–13] and the accurate classification of NEs depends significantly on extracting the NEs correctly. However, the complexities in extracting and classifying NEs vary across languages.

There are multiple challenges when developing NER systems. One of them is the ambiguity of NE types. For example, Sydney may refer to a girl’s name, a city in Australia, or an organization’s name. Differentiating between similar entity types can be difficult and addressing these ambiguities requires contextual understanding and domain knowledge. Moreover, identifying the correct boundaries of named entities within the text can be challenging, especially when dealing with entities that consist of multiple words or have non-standard spellings. Additional challenges include handling nested entities, where an entity is nested in another entity e.g., “Alan Smith, CFO of Hilton Corp,” and out-of-vocabulary entities that are not present in the training data. To build accurate and robust NER systems, addressing these semantic and syntactic challenges by incorporating linguistic knowledge and leveraging contextual information along with domain-specific knowledge and carefully annotated training data.

Urdu language is spoken by 100 million people globally and is one of the widely used languages in the South Asian region [14]. It is written from right to left using Arabic script. The Urdu language is rich in morphology that contributes to the generation of many surface forms of the base words which may occur in a highly articulated and derivative manner. Moreover, the free word order characteristic of the Urdu language allows one to write similar-meaning sentences in more than one word order. Additionally, many other common challenges such as lack of capital letters and scarcity of linguistic resources are faced when processing the Urdu language. Considering the NER task, the Urdu language poses immense challenges due to minimal textual resources and exhaustive language complexities [15].

Existing studies in Urdu NER mainly use three approaches; (1) rule-based, (2) machine learning and deep learning, and (3) hybrid approach. In the rule-based approach, text matching is performed through predefined grammatical rules or manually built gazetteers. The drawback of the rule-based approach is the non-portability of developed rules across various domains as they are domain-specific. Developing rules for multiple domains requires manual effort with domain knowledge and language expertise which can be cumbersome [16].

Machine Learning (ML) based studies use supervised learning techniques that can easily be deployed across domains and hence require comparatively less development effort [17]. Various studies have used models including Conditional Random Fields (CRF) [18], the Hidden Markov Model (HMM) [19], and the Maximum Entropy Model (MEM) [20] etc. These studies outperform the rule-based approach but the success of these models heavily depends on the selection of the features that are derived from the annotated corpus and are used in the training process. Deep learning (DL) based studies use different pre-trained word embedding techniques [21–27] to map the words in vectors using the language vocabulary to automatically extract meaningful relationships among words in the dataset. Due to limited vocabulary size, out-of-vocabulary words pose significant challenges for morphology-rich languages [28] like Urdu due to language complexities [29]. Existing studies have used DL models for Urdu NER [29–31] by utilizing Word2Vec, Txt2Vec, MKW2v, and GloVe embeddings. The hybrid approach [32] uses a combination of rule-based and ML and DL approaches that perform well in domain-specific scenarios. However, this approach requires language-based knowledge and crucial features that are essential for supervised ML-based models to improve the overall performance of the NER task. Additionally, the size and the contextual richness of the training dataset also play an important role in the performance of ML and DL-based models.

Considering the literature on NER for various languages, FastText and Floret word embeddings have shown a significant impact on the performance of the NER task. These embeddings utilize sub-word information that solves the out-of-vocabulary word problems [33–38]. Consider for example the word “unhappiness” which is split into sub-word units such as “un-”, “happi-”, and “-ness”. These embeddings can capture that “un-” denotes negation or reversal, “happi-” represents happiness or positive sentiment, and “-ness” indicates the quality or state. The embeddings reflect the semantic properties of each sub-word unit and how they combine to form the overall meaning of “unhappiness”.

In this study, we propose a deep learning approach for Urdu NER that utilizes FastText and Floret word embeddings using various Recurrent Neural Networks (RNN) configurations. Using four publicly available and widely used Urdu benchmark datasets, we show that our proposed approach significantly outperforms existing state-of-the-art studies on Urdu NER. The main reason for choosing these RNN configurations is their ability to capture long-term dependencies and their suitability for Urdu NER tasks. The main contributions of this study are as follows:

- A deep learning approach for Urdu NER is proposed that utilizes FastText and Floret word embeddings to capture the contextual information of words by considering the surrounding context of words for improved feature extraction.
- Various combinations of Long Short-Term Memory (LSTM), CRF, Gated Recurrent Unit (GRU), and Bidirectional LSTM (BiLSTM) are evaluated on four publicly available benchmark datasets.
- Using precision, recall, and F-score evaluation parameters, the results show that our approach significantly outperforms existing state-of-the-art DL-based NER approaches for the Urdu language achieving a low classification error rate.

The rest of the paper is structured as follows. Section “Challenges of Urdu Named Entity Recognition” describes the challenges of NER for the Urdu language followed by the Section “Literature Review” which presents the literature review. Section “Research Methodology” explains the research methodology of this study. Section “Experiment Setup” presents the experimental setup. Section “Results” presents the results and discussion followed by the conclusion and future work in Section “Conclusion and Future Work”.

## Challenges of Urdu Named Entity Recognition

Like many other South Asian languages, Urdu is also a morphologically rich language that poses many challenges when performing NER tasks for the Urdu language. This is because the context and the semantics of a word depend on its neighboring words. A single word can exist in multiple forms resulting in multiple NEs when used in different contexts. Consider for example the following two sentences:

“مقبول بہت مشہور ہے”

*Maqbool is very famous.*

“علامہ اقبال مقبول شاعر تھے”

*Allama Iqbal was a famous poet.*

The common word in both sentences is “مقبول” -/Maqbool which can either be used as a common noun or a proper noun in the Urdu language.

Additionally, nested NEs are common in the Urdu language where NEs are either inside of another entity or are connected to another entity causing ambiguities in the correct identification of NEs. For instance, in “کراچی یونیورسٹی” -/Karachi University, Karachi is a location that causes a problem in correctly identifying the NE label.

Furthermore, in English and other Latin-based languages, capitalization is used as a key indicator for the NER task as capital letters can exclusively be used to identify the NEs from the text [39, 40]. However, that is not the case with the Urdu language making it difficult to perform NER tasks.

Moreover, English abbreviations are usually used in the Urdu language in a special form [41] causing ambiguity for the NER system to identify and classify these abbreviations. For example, “ایل ڈی اے” -/LDA is an abbreviation of an organization named Lahore Development Authority. The NER system cannot correctly label the word as an Organization NE as it is oblivious to the English dictionary.

The agglutination nature of the Urdu language means that the prefix, lemma, and suffix are added to the root (stem) word with multiple different combinations making a more complicated word structure (morphology) [42]. A token may either change a word’s NE type or the word may not be classified as NEs when agglutinated with other words. For instance, the word “لاہور” -/Lahore is the root form of the word “لاہوری” -/Lahori. “لاہور” -/Lahore is a city of Pakistan while “لاہوری” -/Lahori is an inflected form of Lahore that represents the people residing in Lahore and cannot be considered as a NE.

Furthermore, in Urdu, a single name can be written with various spelling variations [39] as some characters have the same pronunciation, known as homophones [43]. This also causes ambiguity for the NER system.

Another challenge in Urdu NER is the typographic errors of certain characters that are often made by the writers due to character similarity or character variance leading to confusion in orthographical representations. Due to inconsistencies in writing styles, the same NEs may be assigned an incorrect entity type [44]. For instance, the word Saif, written as “صیف” and “سیف”, may get different NE labels due to the writing variations.

## Literature review

This section discusses the existing studies on NER for the Urdu language that mainly use three approaches; (i) rule-based, (ii) ML and DL-based, and (iii) hybrid approach.

### Rule-based approach

Singh et al. [16] propose a rule-based NER system for the Urdu language, for thirteen NE types, using handcrafted rules to extract each type of NEs using dictionaries, lexicons, and affixes lists. Such rules are specified for each class of NEs that rely on domain-dependent gazetteers [45, 46] and syntactic-lexical patterns [47]. Twelve of the NEs tags are taken from the IJCNLP dataset [48] and the thirteenth NE tag is the Izaafats used in the Urdu language. The performance of their system is evaluated on two datasets consisting of news articles from BBC Urdu, showing precision, recall, and F-score of 86%, 90%, 88%, and 58%, 62%, and 60% respectively, for the two datasets. The overall accuracy of the whole system is 74.09%. The limitation of this study is that it uses a fixed window size for NE tags i.e. a maximum of three words can be considered while matching words for NE tags. For NEs consisting of more than three words are misclassified as two or more separate NEs. Another limitation is that the system is unable to handle all date formats. Dates written in the form of multiple words are assigned separate NEs for each word, instead of treating it as a single NE. The system offers no support to handle the segmentation problem in the Urdu language which commonly occurs due to differences in writing styles.

Riaz et al. [39] use the rules specified for six NE types including person name, organization, location, numbers, date, and designation. They evaluate their approach on 2,262 documents taken from the Becker-Riaz corpus [49] that contain news stories. Their rule-based approach outperforms the statistical learning models on the Becker-Riaz corpus by identifying 171 correct NEs out of 187 NEs achieving precision, recall, and F-score of 90.7%, 91.5%, and 90.7% respectively. The proposed NER system does not provide results of individual NEs and lacks details about nested NEs. Furthermore, the defined rules are corpus-dependent and cannot perform well on text other than the Becker-Riaz corpus. Additionally, the system is unable to handle rare words as it is heavily dependent on the specially crafted gazetteer.

Developing an efficient rule-based NER system that can outperform various statistical approaches for the Urdu language requires more time and resources [16, 39, 50].

### ML and DL based approach

Ekbal et al. [51] present a NER study on five South Asian languages including Urdu using language-independent and dependent features for model training, including contextual features, orthographic features, and infrequent words. For Urdu, NEs lists are used and CRF is applied on the IJCNLP dataset [48], reporting an F-score of 35.52%. The major limitation of this study is that it only performs well for the Bengali language and also lacks comparison with other similar existing systems. Mukund et al. [52] deploy an Urdu language information extraction system that comprises a NER sub-module that uses Maximum Entropy (MaxEnt) and CRF for the identification of three types of NEs used in this study. The MaxEnt model is trained and tested on the IJCNLP dataset showing an accuracy value of 68.9% for CRF. The limitation of this study is that the segmentation technique presented for the Urdu language is tested on very small data of only 50 sentences. Furthermore, during the transliteration process between English and Urdu, the authors reported the issue of missing diacritics which can significantly reduce the performance of their approach.

Malik et al. [19] implement HMM for twelve NE types with BIO2 (Beginning, inside, and other) and IOE2 (inside, end tag, other) tagging schemes achieving an F-score of 44.89% and 45.52% respectively, for both tagging schemes on the IJCNLP dataset. They further enhance the performance with the IOE2 tagging scheme by utilizing syntactic rules, name lists, and POS information achieving an improved F-score of 69.12%. However, the POS tagger used lacks accuracy due to differences in writing styles. Additionally, the study does not provide detailed information about the gazetteers used by the NER system. In another study, Malik et al. [53] employ HMM and Artificial Neural Networks (ANN) on the KPU corpus for three NE types. For HMM, unigrams, bigrams, and trigrams are used for training purposes, while word embeddings (word2vec) with window sizes (3 and 5) are used as contextual features for ANN. F-scores of 66.90% and 84.17% are achieved for HMM and ANN respectively. This study only uses one single type of word embeddings for text representation and only three types of NEs in the dataset. The experimentation also lacks performance comparison between other variants of ANNs or with existing state-of-the-art Urdu NER systems.

Authors of [20] use the MaxEnt model on the IJCNLP dataset [48] applying different features such as contextual features of the target word, unigram, and bigrams. They predict rare words by considering character-level affixes and achieve an F-score of 92% and 77.93% on test data and unknown words respectively. However, this study lacks error analysis, results validation, and comparison with existing state-of-the-art studies. Wahab et al. [18] apply a supervised CRF approach to the IJCNLP dataset for seven NE types. They use the existing CRF-based approach as a baseline and use the same features on the newly developed UNER dataset [44]. They evaluated the performance for both datasets and achieved f-scores of 73.19% and 74.81% for the baseline and proposed approach respectively. The comparison with existing state-of-the-art studies on Urdu NER is not reported.

The DL-based approach is the most recently adopted technique for Urdu NER tasks. Kanwal et al. [30], generate six-word embeddings by using three existing word embedding techniques as features and use two DL approaches i.e., CNN and Recurrent Neural Network(RNN). The results are compared with CRF and MaxEnt supervised learning models. For CRF and MaxEnt, all techniques are evaluated on MKPUCIT [30] and IJCNLP [48] datasets with ten-fold-cross-validation. A total of 32 experiments are performed. Results show that RNN is more capable of capturing the sequential data and outperforms CNN, while CRF and MaxEnt showed an F-score of 44% and 49%, respectively. The MKPUCIT dataset used in this study only contains three NE types and a limited number of NEs. A thorough review of the dataset shows that a large number of NEs are labeled incorrectly which can have a significant impact on the performance.

Wahab et al. [31], apply Deep Recurrent Neural Network (DRNN) models, with word embeddings used as features, for seven NE types namely, person, organization, location, date, time, designation and, number. IJCNLP [48], Jahangir et al. [54] and UNER [44], datasets are used for performance evaluation using word-level, language-dependent and, language-independent features. Their DNN model outperforms traditional approaches on all three corpora with F-scores of 81.1%, 79.9%, and 63.21% respectively. Authors in [55, 56] apply the DL approaches on the MKPUCIT dataset [30]. Three pre-trained embeddings i.e., word2vec, Glove, and FastText are generated and input to BiLSTM. The output of BiLSTM is passed to the self-attention layer and then the dense layer. CRF is used as the final output prediction for the UNER task. The performance of word2vec outperforms glove and FastText word embeddings with an F-score of 93% on the MKPUCIT dataset.

One of the limitations of applying ML and DL to NER system development is the availability and the contextual richness of the dataset. Moreover, the size of the dataset also plays an important role that can lead to underfitting or overfitting of the model.

### Hybrid approach

Due to the limited availability of linguistic resources, hybrid approaches are used to develop the NER system for the Urdu language to overcome various challenges.

Mukund et al. [32] apply the CRF approach for the identification of three NE types namely, person, organization, and location. Words with POS information are used by following the bootstrapping methods to increase the size of training data. A four-stage model is adopted in which the first three stages are trained for the CRF approach and one stage uses the rule-based approach to maximize the performance of NEs tagging. For all stages of training, unigram and bigram features for the tags and words are used. The performance is evaluated on the CRULP dataset [50] with ten-fold cross-validation achieving an F-score of 68.9%. The system lacks correct labeling of PERSON and LOCATIONS tags due to ambiguities in the rules crafted for tagging.

Gali et al. [57] use CRF along with a rule-based approach for five languages to identify twelve NE types. The traditional supervised CRF approach is also used with various features such as contextual, orthographic, morphological, and word-level features along with the POS information to improve performance. The proposed approach achieves an F-score of 43.4% for the Urdu language. A small data size is used for the Urdu language with no dataset information except the total number of tokens. Further, the comparison with other similar studies is missing to evaluate the strength of the proposed approach. Saha et al. [58] present a hybrid NER system by combining the hand-crafted rules with the Max-Ent model for five South Asian languages. The proposed system is evaluated on the IJCNLP dataset for twelve NE types, achieving an F-score of 35.47%. In this study, the authors used rules for Hindi and Bengali languages, but Urdu, Oriya, and Telugu were trained and tested on a simple Max-Ent model.

Domain expertise and language knowledge are required to develop the rules for rule-based approaches which can be a cumbersome task. For the traditional ML-based approach and hybrid approach, manual feature extraction requires large, labeled datasets which do not usually exist for scared-resource languages like Urdu. However, DL-based approaches extract features from the dataset automatically using techniques such as word embeddings. Table 1 presents the literature review on Urdu NER.

**Table 1:** Literature review on Urdu NER.

Ref.	Approach	NEs	Attributes	F-score
2012 [16]	Rule-based	13	Orthographic and gazetteer-list	0.88
2010 [39]	Rule-based	6	Lexical indication and gazetteer-list	0.91
2022 [18]	ML-based	7	Language-dependent, and language-independent features	0.74
2017 [19]	ML-based	12	Part of Speech (POS) information, gazetteers, and rules	0.69
2020 [20]	ML-based	12	Contextual features, unigram/bigrams & rare words prediction by character level affixes	0.92
2008 [51]	ML-based	12	Contextual, orthographic features, infrequent words, case mechanism, list of gazetteers	0.35
2010 [52]	ML-based	3	Words along with POS information	0.69
2012 [54]	ML-based	5	Gazetteer-List and orthographic feature using smoothing techniques	0.76
2017 [53]	ML/DL-based	3	Orthographic and contextual features	0.84
2019 [30]	DL-based	3	Word embeddings	0.49
2020 [31]	DL-based	3	Word embeddings, language-dependent, and language-independent features	0.81
2022 [55]	DL-based	3	Word2vector, FastText and Glove used as features	0.93
2022 [56]	DL-based	3	Word embeddings using embedding-level focus mechanism	0.92
2009 [32]	Hybrid	3	Words with POS information	0.69
2008 [57]	Hybrid	12	contextual, orthographic, morphological, and word-level features with linguistic rules	0.45
2008 [58]	Hybrid	12	Language-independent features and rules	0.48

## Research methodology

**Fig 1:**
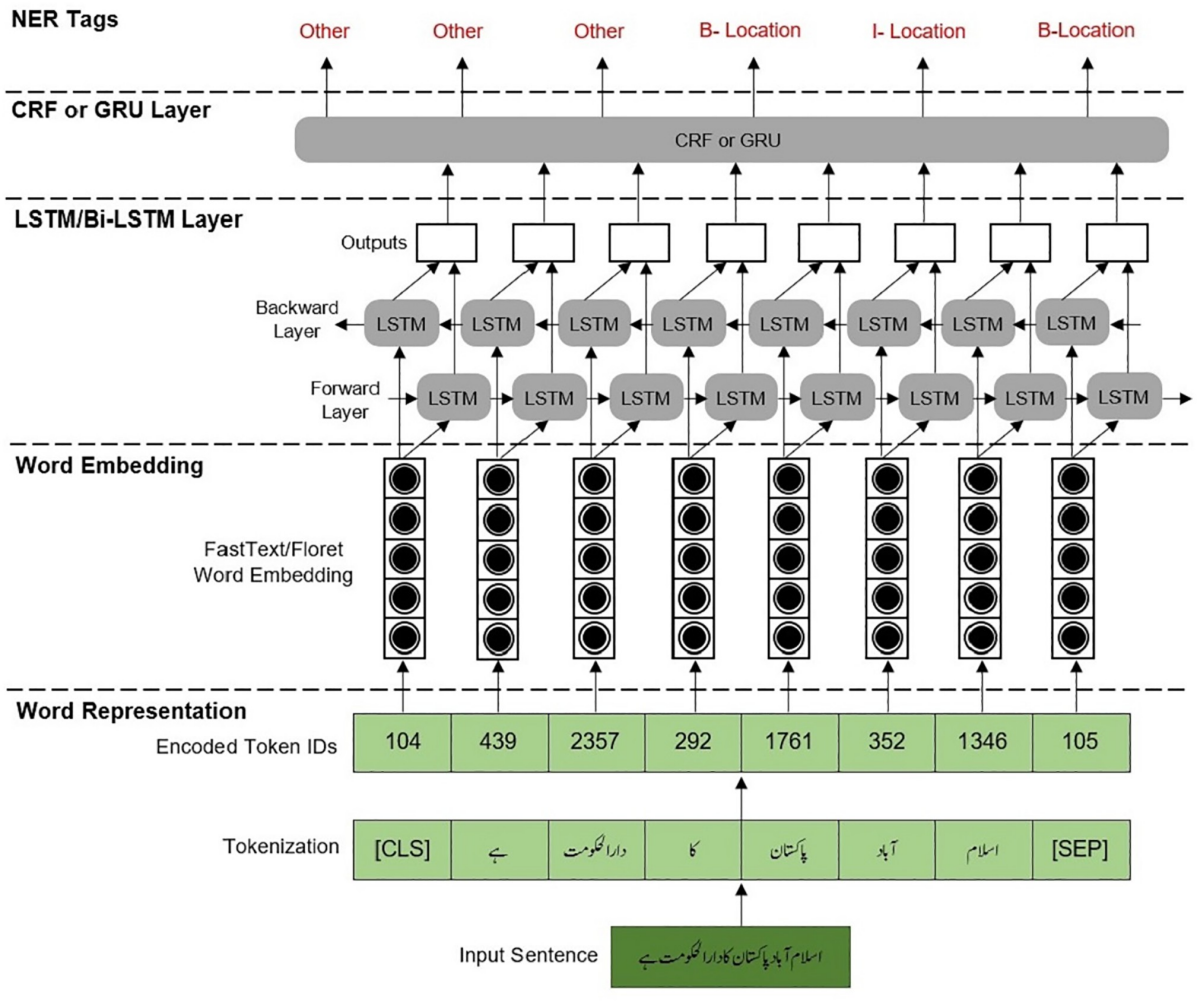
Methodology of the proposed Urdu NER system.

This section explains the methodology used by the DL-based Urdu NER system proposed in this study. A sentence is initially passed to our Urdu NER system which is tokenized into words. These words are then encoded and passed to the pre-trained FastText and Floret word embeddings for feature extraction that converts these encoded words into feature vectors which are then input to the DL models for feature classification. For each dataset, we use six combinations of DL models, (i) LSTM, (ii) BiLSTM, (iii) LSTM followed by CRF (LSTM-CRF), (iv) BiLSTM followed by CRF (BiLSTM-CRF), (v) LSTM followed by GRU (LSTM-GRU), and (vi) BiLSTM followed by GRU (BiLSTM-GRU). The proposed methodology of our Urdu NER system, illustrated through an example sentence, is shown in Fig 1.

Using FastText and Floret word embeddings, the proposed system helps address various challenges of Urdu NER discussed in the previous section. These word embeddings provide contextual support among various words of a sequence, helping capture semantic and contextual information about words based on their surrounding words. This helps in addressing the challenge of words having multiple forms in different contexts by assigning the appropriate NE labels based on the neighboring words. Additionally, this contextual representation also helps in representing the relationship between nested words thereby significantly reducing the ambiguities in identifying nested NEs. The challenge of homophones and typographical errors are also addressed through contextual information. For the correct classification of English abbreviations, our system removes the special characters and unnecessary spaces in the pre-processing phase for uniform presentation before generating word embedding. FastText and Floret embeddings, which are designed to provide a fine depiction of abbreviations that may occur in special forms in the text, help capture the special forms of English abbreviations used in Urdu text by learning the representations of such abbreviations even if they are not present in the English dictionary. FastText and Floret word embeddings address the challenge of agglutination, which occurs due to adding prefixes, lemmas, and suffixes to a root, by comprehending the morphological variations in words and encoding information about the root of a word and its derived words thereby precisely representing complex word structures, which is crucial for accurate NE recognition.

### Datasets and preprocessing

To evaluate the proposed Urdu NER system in this study, we use four publicly available and widely used benchmark datasets namely IJCNLP [48], Jahangir et al. [54], MKPUCIT [30] and UNER [44] that cover a large number of NEs. The IJCNLP dataset was the first publicly available dataset for the Urdu language comprising six tagged NEs namely, person, organization, location, designation, time, and Number. Jahangir et al. [54] developed a tagged dataset containing 1,526 unique NEs of five types namely, person, organization, location, time, and date. MKPUCIT dataset [30] is the largest dataset generated for Urdu NER tasks comprising three types of NEs namely, person, organization, and location, and is an extension to the previous KPU-NE corpus. The data is extracted from Newslink.pk website. MKPUCIT dataset is significantly different in terms of size, domain areas, NE classes, and dataset distribution which may affect the performance of the NER task. UNER dataset [44] is developed by collecting data from news articles from the BBC Urdu website. It is tagged for seven NE types namely, person, organization, location, designation, time, number, and date, and is a well-structured dataset for Urdu NER tasks, requiring minimum preprocessing effort. Different statistics of these datasets are shown in Table 2.

**Table 2:** Summary of the used datasets.

Datasets	Tokens Count	Sentence Count	Unique NEs	NE Types
IJCNLP	40,408	1,097	1,115	6
Jahangir	31,860	1,315	1,526	5
MK-PUCIT	926,776	36,215	99,718	3
UNER	48,673	1,744	4,621	7

Some preprocessing was required before we could use the datasets for experimentation including the addition and removal of spaces between tokens for the improved structure of the multi-word NEs, removal of punctuation marks, filtering duplicate words, handling entangled words with labels, and handling of single letters. The count of NEs is sufficient in the datasets but in some datasets, the data was not equally distributed causing class imbalance over different NE types. The class imbalance was also addressed in the preprocessing phase. In some datasets, some classes contained a very low number of instances. This was handled by applying oversampling to increase the number of instances in minority classes by replicating random instances from these classes to balance them with the rest of the classes. For the datasets used in this study, this was only required for the “designation class” in the IJCNLP and UNER datasets.

### Feature extraction

The DL models used for the NER task require numeric data, in the form of feature vectors, which are used for the classification of NEs. The traditional method of generating the feature vector is to use a one-hot vector that represents unique words with ‘0’ for the non-target word and ‘1’ for the target word. The problem with these representations is that they cannot build relationships among words. Existing studies [55, 56, 59] have shown that using word embeddings outperforms manual feature selection for the NER tasks. Word2vec [21] is a widely used word embedding that incorporates the information of skip-grams or continuous bag-of-words. While word2vec outperforms traditional methods of generating word embeddings, it still lacks information such as n-grams. FastText and Floret word embeddings have shown a significant impact on the performance of the NER tasks for various languages [33–38]. These embeddings utilize sub-word information that solves the out-of-vocabulary word problems. For feature extraction, in this study, we use FastText and Floret word embeddings that are generated for all pre-processed datasets by using the pre-trained embedding files for the Urdu language.

Datasets used in this study lack POS information making it crucial to correctly label nested NEs. The challenge of nested NEs is addressed by employing word embeddings in the proposed approach. Both Floret and FastText embeddings utilize sub-word information, by breaking each word into possible sub-words that help to capture morphological leads to create contextual relationships among words. This is essential to correctly label nested NEs, as the meaning of a sub-word commonly depends on the larger word associated with it. For instance, in the sentence “شاہ جہاں نے تاج محل آگرہ میں بنوایا، جو مغل بادشاہوں کا مقبرہ ہے” -/Shah Jahan built the Taj Mahal in Agra, which is the tomb of the Mughal emperors, “تاج محل آگرہ” -/Taj Mahal Agra is a nested entity within “مغل بادشاہوں کا مقبرہ” -/tomb of the Mughal emperors. Embeddings capturing sub-word information and context could help identify both entities accurately.

#### FastText embeddings

FastText embeddings, developed by Facebook research group [60], primarily work with the morphology of the words. FastText, an extension of word2vec, uses n-grams to generate multiple variations of a given word by breaking it into sub-words depending on the given window size. The resultant vector of the input word is the sum of all n-grams generated for that word. This helps to improve the representation of rare words in the input data. FastText embeddings play a significant role in morphologically rich languages such as Urdu and Arabic where a single word may have various morphological forms and each of them may occur rarely. Although FastText embedding improves the capability of the NER system to understand text semantics, its drawback is that for datasets having a large vocabulary, it generates vectors of considerable length that are difficult to process by the DL models.

#### Floret embeddings

Floret embeddings [61] is an extended version of FastText that captures the same information as FastText but stores it differently by reducing the size of the vector up to ten times comparatively. In FastText, words and character n-grams are stored separately, and the word table contains one entry of a single word in the vocabulary while character n-grams are stored in a separate fixed-size table by hashing the one character per row in the table. Floret compresses the size of the vector table by storing the words and their character n-grams into the same hash table having more than one entry in each row which helps to reduce the size of the hash table. These multiple hashes are unlikely to collide with other hashes, as most of the entries will be distinct vector representations. The FastText and Floret n-gram window size defines the amount of information they can capture from the given text. For this study, we tested different n-gram window size values ranging from three and six and found that five is the optimal n-gram window size value as it helps to capture the necessary contextual information effectively.

### Feature classification

For feature classification, the feature vectors generated by the word embeddings are input to different combinations of DL models, using LSTM, BiLSTM, GRU, and CRF, as discussed previously. LSTM/BiLSTM is used as the first layer of the proposed architecture while GRU/CRF is used as the second layer of the proposed architecture. BiLSTM processes input sequences in both forward and backward directions by capturing the contextual information from past and future words as the context of a word influences its entity type. The output of the BiLSTM layer is passed to the CRF layer. CRF is a probabilistic graphical model often used for sequence labeling tasks, as it considers dependencies between labels and performs global inference to predict the most likely sequence of entity labels for the inputs. When used in combination with BiLSTM, CRF helps in modeling the transition probabilities between different entity labels, improving the coherence of the predicted labels. The combination of BiLSTM and GRU is also adopted which is effective in predicting labels.

#### LSTM/BiLSTM

Recurrent Neural networks (RNN) have been applied to various classification problems, including Named entity recognition [62], which outperform traditional machine learning approaches. However, RNNs are unable to hold information for long sequences of data making it difficult to transfer information from the initial steps to the later steps, also known as the vanishing gradient problem. LSTM [63] is the type of RNN that solves the vanishing gradient problem through the gated structure that can retain long-term dependencies between the input data by shifting the relevant information down the chain of sequences to make predictions. In LSTM cells, the flow of information is controlled through input, forget, and output gates, in combination with the activation functions. The input gate decides which information is needed during the current step. An input layer represents the features at a given Time (t) and has the same dimensional size as that of the feature size. The forget gate determines the necessary information that needs to be kept from previous steps. An output layer is the probability distribution over the NEs labeled at time (t). LSTM also has a memory-built cell state and a hidden state that holds the relevant information which is formulated as:

$$\begin{array}{c} i_{t}=\sigma \left(W_{i}[h_{t-1},x_{t}]+b_{i}\right) \end{array}$$ (1)

$$\begin{array}{c} f_{t}=\sigma \left(W_{f}[h_{t-1},x_{t}]+b_{f}\right) \end{array}$$ (2)

$$\begin{array}{c} O_{t}=\sigma \left(W_{o}[h_{t-1},x_{t}]+b_{o}\right) \end{array}$$ (3)

$$\begin{array}{c} C2_{t}=tanh\left(W_{c}[h_{t-1},x_{t}]+b_{c}\right) \end{array}$$ (4)

$$\begin{array}{c} C_{t}=f_{t}\cdot C_{t-1}+i_{t}\cdot C2_{t} \end{array}$$ (5)

$$\begin{array}{c} h_{t}=O_{t}\cdot tanh\left(C_{t}\right) \end{array}$$ (6)

where *i*_*t*_, *f*_*t*_, *O*_*t*_ and *C*_*t*_ represent input gate vectors, forget gate vectors, output gate vectors, and cell vectors respectively, having hidden state *h*_*t*_ of the same size. *σ* denotes the activation function, *b* and *W* are the connection weights that are computed during the training.

In an NER task, the current position of a word can be tagged correctly if the model can acquire the past and future position of the word information at a given point in time. LSTM can process long text, but the model can just acquire past information. BiLSTM allows getting more contextual information by using the two LSTMs, one taking the input in the forward direction and the other going in the backward direction of the given sequence [64]. The final representation of the word concentrates on the left and right context making efficient use of previous and future features for a given time frame. BiLSTM helps capture information on NEs with specific prefixes and suffixes. In this study, we use a total of 300 vector dimensions for each word, 150 each for the backward and forward pass.

#### Gated recurrent unit

GRU is a variation of LSTM with an addition of two gates namely, update gate *y*_*t*_ and reset gate *r*_*t*_ that control the long-term dependencies as it lacks the output gate [65]. The update gate consists of an input gate and a forget gate that retains the relevant data and passes it to the next units. A reset gate finds the relevancy of the previous cell state which finds the next candidate. The cell state is equivalent to the hidden state through the *tanh* layer creating a new candidate vector *h*2_*t*_ using a reset gate.

$$\begin{array}{c} y_{t}=\sigma \left(W_{y}[h_{t-1},x_{t}]+b_{y}\right) \end{array}$$ (7)

$$\begin{array}{c} r_{t}=\sigma \left(W_{r}[h_{t-1},x_{t}]+b_{r}\right) \end{array}$$ (8)

$$\begin{array}{c} h2_{t}=tanh\left(W_{c}[r_{t}\cdot h_{t-1},x_{t}]+b\right) \end{array}$$ (9)

$$\begin{array}{c} h_{t}=\left(1-y\right)*\cdot h_{t-1}+y_{t}*h2_{t} \end{array}$$ (10)

In the above equations, *y*_*t*_ represents the update gate, *r*(*t*) is the reset gate, *σ* is an activation function, *h*_*t*_ is the hidden state, *x*_*t*_ is input cell vector whereas b and w are the parameters.

#### Conditional random fields

To consider the sentence level tag in the NER task, CRF is used as the prediction layer which takes the preceding word and the succeeding word label which helps predict the correct labels in the testing phase. The input and output are connected through neural layers where either memory cells or recurrent units are utilized. In the output layer, the sequence of the label given as input to the CRF layer calculates the conditional probabilities of every possible label sequence of a given word, helping improve the tagging accuracy of a model by acquiring the contextual information of the neighboring words’ corresponding labels [64].

## Experiment setup

In this study, we use four publicly available benchmark datasets, namely IJCNLP [48], Jahangir et al. [54], MKPUCIT [30] and UNER [44]. For each dataset, we conducted 12 experiments, making it a total of 48 experiments. For the FastText and Floret embeddings, we deploy six combinations of DL models, namely LSTM, LSTM-CRF, LSTM-GRU, BiLSTM, BiLSTM-CRF, and BiLSTM-GRU, for training purposes. Experiments are conducted on a system having 64 GB of RAM, GTX 1080 Ti GPU, and Intel Core i7 8700K processor. The word embeddings are generated against each dataset using a custom Python script and vectors are stored in text files. The DL model training is performed using Keras, a python-based library developed by Google which is widely used to perform various NLP-related tasks. A split ratio of 70–20-10% is used for each dataset for the training, testing, and validation.

### Hyperparameter tuning

The optimal selection of parameters significantly impacts the overall performance of the DL models. Based on prior knowledge and after performing multiple experiments, the values of different parameters are fine-tuned. The best-performing parameter settings, used in this study, are shown in Table 3. The word embeddings dimension is set to 300 and a dropout rate is set to 0.5 for different neural layers. Adam optimizer is used with a batch size of 32 and a learning rate of 0.001. The “ReLU” optimizer is used with a time-distributed layer that helps balance the output dimensions. To improve the performance of LSTM and BiLSTM, in some configurations, regularization is performed by setting a dropout rate of 0.5, which also helps to avoid overfitting the model while training. Determining the optimal n-gram window size depends on multiple factors including the nature of the text in a corpus, different sentence structures that may produce different patterns and dependencies between words, and target language and writing styles. For the Urdu language, various studies generally use an n-gram window size ranging from 3 to 5 for different NLP tasks while existing studies on Urdu NER [29–31, 53] have used a window size of five. For this study, we tested different n-gram window size values ranging from three to five and selected the best-performing n-gram window size of five. For this study, we use an epoch size of 10.

**Table 3:** Summary of hyperparameter tuning.

Parameters	Values
Hidden layers	3
Hidden units	300
Epochs size	15
Learning rate	0.001
Batch size	32
Optimization technique	Adam
Embedding dimensions	300
Dropout rate	0.5
Activation functions	Softmax, Relu
Validation split	0.1
Loss function	Categorical Crossentropy
n-gram (window size)	5
Vector dimensions	300

### Evaluation metrics

We evaluate our proposed Urdu NER system using the precision, recall, and F-score evaluation measures, calculated using the equations given below. The precision measure shows how precisely a model is recognizing the instances and is the ratio of the number of instances correctly identified as positive to the total number of identified instances. Recall is the ratio of the number of instances correctly identified as positive to the total number of true positive identified instances. F-score is the harmonic mean of precision and recall.

$$\begin{array}{c} Precision\left(P\right)=\frac{T_{p}}{T_{p}+F_{p}} \end{array}$$ (11)

$$\begin{array}{c} Recall\left(R\right)=\frac{T_{p}}{T_{p}+F_{N}} \end{array}$$ (12)

$$\begin{array}{c} F-Score=2\times \frac{\left(P\times R\right)}{\left(P+R\right)} \end{array}$$ (13)

## Results

Table 4 shows the results of our Urdu NER system of four publicly available benchmark datasets of the Urdu language namely IJCNLP [48], Jahangir et al. [54], MKPUCIT [30], and UNER [44] that cover a large number of NEs. The summary of these datasets is shown in Table 2. Six different combinations of DL models with FastText embedding and Floret embedding are evaluated. Each dataset contains different types of NE labels that are evaluated individually for all six DL model configurations. Since in some datasets, the data is not equally distributed causing a class imbalance over different NE types, the macro average of class-wise scores of each NE type is reported as it considers each class equally. The results of each class are computed individually which are then aggregated. This ensures that the evaluation is representative of the model’s ability to classify all classes rather than being dominated by the performance of the majority class.

**Table 4:** Results with different deep learning configurations.

Datasets	Model	FastText Embeddings			Floret Embeddings		
	Configurations	Precision	Recall	F-score	Precision	Recall	F-score
IJCNLP [48]	LSTM	0.96	0.92	0.94	0.97	0.94	0.95
	LSTM-CRF	0.96	0.94	0.95	0.97	0.93	0.95
	LSTM-GRU	0.96	0.93	0.95	0.96	0.94	0.95
	BiLSTM	0.97	0.95	0.96	0.96	0.96	0.96
	BiLSTM-CRF	0.97	0.94	0.95	0.97	0.96	0.96
	BiLSTM- GRU	0.97	0.93	0.95	0.96	0.97	0.97
Jahangir [54]	LSTM	0.96	0.93	0.94	0.95	0.95	0.95
	LSTM-CRF	0.92	0.90	0.91	0.95	0.95	0.95
	LSTM-GRU	0.95	0.95	0.95	0.95	0.95	0.95
	BiLSTM	0.96	0.95	0.96	0.95	0.96	0.96
	BiLSTM-CRF	0.88	0.79	0.79	0.95	0.97	0.96
	BiLSTM- GRU	0.95	0.95	0.95	0.95	0.97	0.96
MKPUCIT [30]	LSTM	0.97	0.97	0.97	0.85	0.79	0.81
	LSTM-CRF	0.96	0.97	0.97	0.81	0.77	0.79
	LSTM-GRU	0.96	0.96	0.96	0.85	0.80	0.82
	BiLSTM	0.95	0.97	0.96	0.84	0.87	0.85
	BiLSTM-CRF	0.90	0.93	0.91	0.59	0.58	0.50
	BiLSTM- GRU	0.97	0.98	0.98	0.83	0.86	0.85
UNER [44]	LSTM	0.94	0.96	0.95	0.96	0.97	0.97
	LSTM-CRF	0.95	0.96	0.96	0.96	0.97	0.97
	LSTM-GRU	0.95	0.95	0.95	0.96	0.97	0.97
	BiLSTM	0.96	0.98	0.97	0.95	0.99	0.97
	BiLSTM-CRF	0.95	0.96	0.95	0.96	0.99	0.97
	BiLSTM- GRU	0.94	0.96	0.95	0.97	0.99	0.98

**Fig 2:**
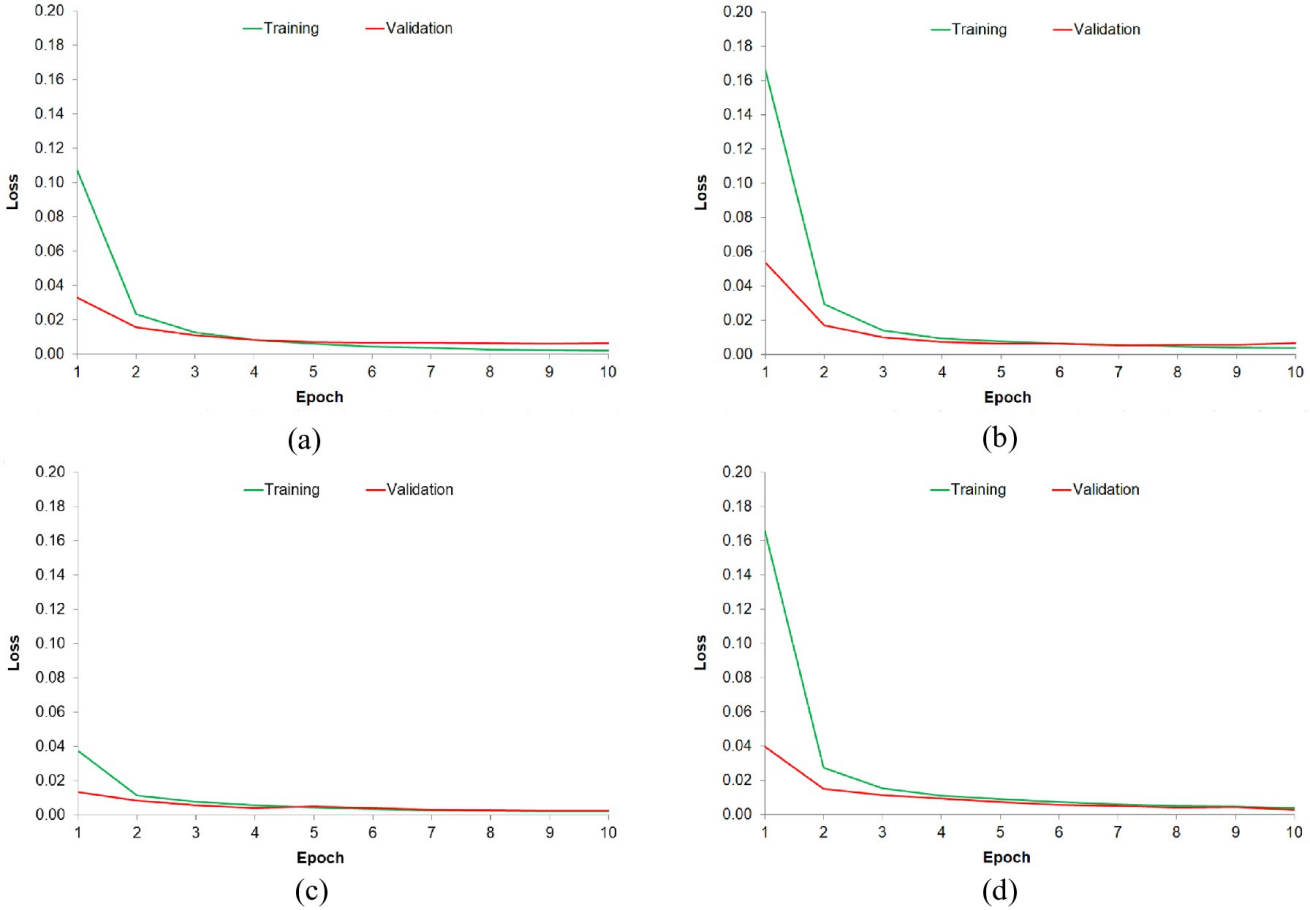
Training and Validation loss for best-performing models (a) BiLSTM-GRU with Floret embedding on IJCNLP, (b) BiLSTM-GRU with Floret embedding on Jahangir, (c) BiLSTM-GRU with FastText embedding on MKPUCIT, and (d) BiLSTM-GRU with Floret embedding on UNER.

The proposed study adopts different variants of best-performing RNN configurations from existing literature as a baseline by starting the experimentation LSTM or BiLSTM layer and subsequently incorporating a GRU or CRF layer on preprocessed benchmark datasets. It is observed from the results that the BiLSTM-GRU combination performed the best on all benchmark datasets. BiLSTM-GRU with Floret embeddings performed better on IJCNLP, Jahangir, and UNER datasets achieving an F-score of 0.97, 0.96, and 0.98 respectively. BiLSTM-GRU with FastText embeddings performed better only on the MKPUCIT dataset, achieving an F-score of 0.98. For the UNER dataset, it is observed that all the DL models performed uniformly with an F-score ranging from 0.97 to 0.98 which indicates that the UNER dataset is the most balanced in terms of NE types as compared to other datasets. BiLSTM+GRU performs better because comparatively, it is a simpler model than BiLSTM+CRF as it does not have a decoding layer which helps reduce the training time making it favorable for large datasets. This can be observed from the results of the MKPUCIT dataset which is the largest dataset used in this study. Floret embedding seems to perform better even for imbalanced datasets that contain infrequent/rare NEs. This is because it captures the contextual information of words that is crucial for NER tasks. By considering the surrounding context, it can understand the context-dependent nature of NEs, improving their recognition. Additionally, rather than relying solely on frequency of occurrence, Floret embedding represents words based on their semantic similarity, which helps in capturing the similarity even for infrequent NEs in the dataset. However, the effectiveness of Floret embedding for NER on imbalanced datasets can vary depending on the specific characteristics of the dataset and the complexity of the named entities involved. Fig 2 shows the training and validation loss of best-performing models on all four benchmark datasets.

### Comparison with state-of-the-art approaches

To evaluate the robustness of our proposed Urdu NER system, we compare our approach with the existing state-of-the-art methods used for Urdu NER against all four benchmark datasets. The comparison is made based on the embeddings used with each type of DL model as shown in Table 5. A performance improvement of 32%, 12%, and 11% in F-score is observed for IJCNLP, Jahangir, and UNER datasets respectively, for our approach using BiLSTM-GRU with Floret embeddings, over existing studies. A performance improvement of 5% in F-score is observed for the MKPUCIT dataset for our approach using BiLSTM-GRU with FastText embeddings, over existing studies. This validates the robustness of our proposed approach as it outperformed all existing state-of-the-art DL approaches. Furthermore, it is also validated that Floret word embeddings performed the best for automatic feature selection in Urdu NER tasks on all benchmark datasets as it captures the contextual information of words crucial for NER tasks and can comprehend the context-dependent nature of NEs by considering the surrounding context of the word leading to better to improved performance. Moreover, Floret embedding represents words based on their semantic similarity leading to capturing the similarity even for infrequent NEs in the dataset.

**Table 5:** Performance comparison of the proposed approach with state-of-the-art on all benchmark datasets.

Dataset	Approaches	Embeddings	Precision	Recall	F-score
IJCNLP	RNN [30]	Word2vec	0.69	0.63	0.65
	LSTM-Foward RNN [31]	Txt2vec	0.53	0.73	0.61
	BiLSTM+CNN [29]	Word2vec + Character	0.65	0.65	0.65
	**Proposed BiLSTM+GRU**	Floret	0.96	0.97	0.97
Jahangir	LSTM-Foward RNN [31]	Txt2vec	0.80	0.81	0.80
	Bi-GRU+CNN [29]	Word2vec + Character	0.83	0.84	0.84
	**Proposed BiLSTM+GRU**	Floret	0.95	0.97	0.96
MKPUCIT	RNN [30]	Word2vec	0.76	0.78	0.77
	Att-BiLSTM-CRF [55]	Word2vec	0.93	0.93	0.93
	Att-BiLSTM-CRF [55]	FastText	0.91	0.93	0.92
	Att-BiLSTM-CRF [55]	Glove	0.90	0.91	0.91
	**Proposed BiLSTM+GRU**	FastText	0.97	0.98	0.98
UNER	LSTM-Forward RNN [31]	Txt2vec	0.85	0.79	0.81
	BI-GRU+CNN [29]	Word2vec	0.87	0.87	0.87
	**Proposed BiLSTM+GRU**	Floret	0.97	0.99	0.98

### Error analysis

**Fig 3:**
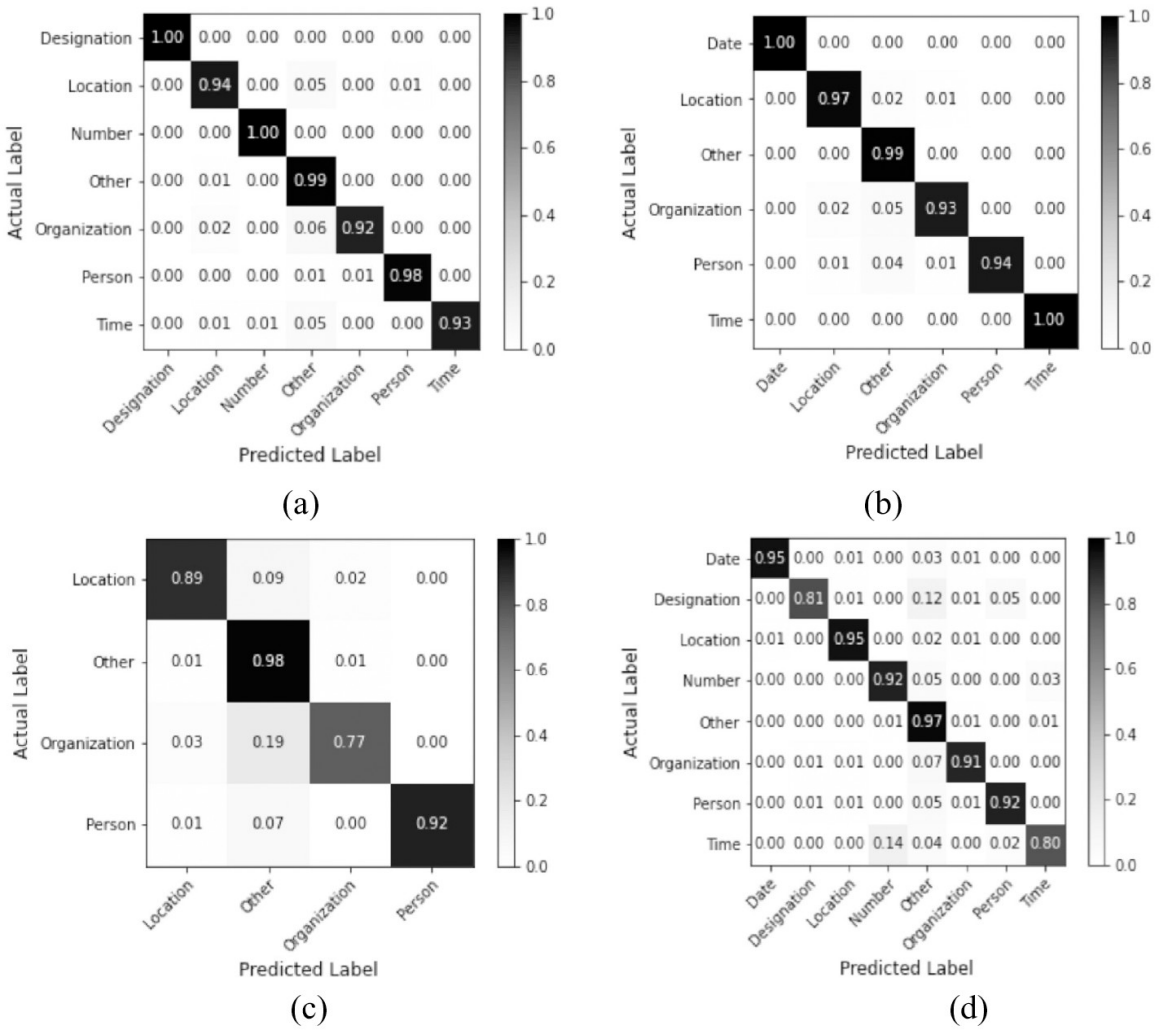
Confusion Matrix of best-performing models (a) BiLSTM-GRU with Floret embedding on IJCNLP, (b) BiLSTM-GRU with Floret embedding on Jahangir, (c) BiLSTM-GRU with FastText embedding on MKPUCIT, and (d) BiLSTM-GRU with Floret embedding on UNER.

Misclassification is a common issue while performing NER tasks in the Urdu language. To analyze the misclassified tokens, a class-wise comparison of actual labels with their predicted label is performed for testing data for best-performing configurations. Fig 3 shows the confusion matrix of best-performing models for all datasets.

Two domain experts manually performed a comprehensive analysis to identify the reasons behind misclassification, and five fundamental causes were found including (1) an insufficient number of uncommon name entities in the training set; (2) single letter words and abbreviations; (3) words from languages other than Urdu; (4) incorrect tokenization; (5) ambiguous multi-word NEs; and (6) incorrect labeling in the training set. It is also observed that the UNER dataset performed best with the least misclassified tokens and an error rate of 1.24%. The highest number of misclassifications occurred on the IJCNLP dataset with an error rate of 3.63% while the MKPUCIT and Jahangir datasets showed error rates of 1.74% and 1.52% respectively. A lower error rate on all benchmark datasets validates the correctness of the proposed technique for the Urdu NER task.

Many words in the Urdu language can have multiple meanings or can be used in various contexts. The proposed model uses Floret word embeddings to capture contextual information. Floret captures the morphological variations of a word and the different labels associated with each variation. For instance, the word “اقبال” -/Iqbal is a person’s name and is classified as PERSON, but “اقبال پارک” -/Iqbal Park is a similar multi-word name entity that is classified as LOCATION. Iqbal Park can produce different meanings if both words are used separately. These word embeddings capture such short-term and long-term dependencies between multiple words and help the model build intuition about the context of the given text. This contextual intuition solves different problems related to name entity disambiguation as each word variation is handled according to the available contextual information from the surroundings.

Since these embeddings capture available contextual information from the surroundings, the performance can vary across the training dataset. As observed in this study, the proposed model sometimes confuses the NEs classes that are labeled in the dataset. For instance, person NEs types were misclassified into organization and location. Similarly, organization NEs types were conflated to person and location. Similarly, location NEs were misclassified with person and organization. These conflations are caused by the aforementioned reasons. For example, “یارخان” -/Yar Khan is a name of a person but it was misclassified as a location because of the existence of “رحيم يار خان” -/Rahim Yar Khan in the dataset which is a city name.

A single word can have different semantics in different contexts. For instance, an example of a location “اسلام آباد” -/Islamabad; the first part “اسلام” -/Islam is classified as a person. The main reason is that an ambiguous entity can be a common noun, a proper noun, or an adjective. We also noted an organization NE is misclassified as a person or a location. For example, “پشاور یونیورسٹی” -/Peshawar University; the first part of this organization is “پشاور” -/Peshawar, which a name of the city and is misclassified to location. Similarly, “پیپلزپارٹی” -/Peoples party is an organization’s name but is conflated to person.

## Conclusion and future work

This study presents a deep learning approach for NER for the Urdu language. The proposed approach utilizes FastText and Floret word embeddings to capture the contextual information of words by considering the surrounding context of words for improved feature extraction. Various combinations of LSTM, BiLSTM, GRU, and CRF deep learning models are used for the classification of NEs. Four publicly available Urdu benchmark datasets are used to evaluate our proposed approach. Results show that the proposed model significantly outperforms existing state-of-the-art studies on Urdu NER achieving an F-score of up to 0.98 when using BiLSTM+GRU with Floret embeddings. This is due to the context sensitivity and task-specific adaptability of Floret embeddings over Word2vec and Txt2vec, used in existing studies. Error analysis is performed between the actual tags and the predicted tags achieving a low classification error rate ranging from 1.24% to 3.63% across various datasets showing the effectiveness and robustness of the proposed technique. Floret embeddings are effective at recognizing NEs that are similar to those seen during pre-training and may struggle for NER tasks involving rare or completely unseen entities as it relies on the pre-training data and the model may not have encountered the specific characteristics or context of those entities during training. This can be addressed by data augmentation, domain-specific embeddings, ensemble learning by combining multiple pre-trained embeddings, and training models on larger and more diverse datasets. Existing datasets are generated by scraping the News websites and the data comprises multiple domains which are split into testing and training data. Our model performs well as in the testing phase it may not encounter completely unseen entities. Therefore, cross-domain NER is a challenging task for existing studies and the proposed approach. In the future, we plan to develop a diverse cross-domain dataset for NER that includes domains of history, science, sports, etc. which contain rich NEs having more NE types, and develop deep learning-based models for cross-domain NER.

## Supporting information

S1 File (ZIP)
